# Freiburg Neuropathology Case Conference: Progressive Optic Nerve Lesion Over a 16-Year Period

**DOI:** 10.1007/s00062-025-01505-8

**Published:** 2025-02-05

**Authors:** I. E. Duman Kavus, R. Sankowski, R. Rölz, A. Dressing, M. Prinz, H. Urbach, D. Erny, C. A. Taschner

**Affiliations:** 1 Department of Neuroradiology, Medical Centre, University of Freiburg, Breisacherstrasse 64, 79106 Freiburg, Switzerland; 2 Department of Neuropathology, Medical Centre, University of Freiburg, Breisacherstrasse 64, 79106 Freiburg, Switzerland; 3 Department of Neurosurgery, Medical Centre, University of Freiburg, Breisacherstrasse 64, 79106 Freiburg, Switzerland; 4 Department of Neurology, Medical Centre, University of Freiburg, Breisacherstrasse 64, 79106 Freiburg, Switzerland; 5 Faculty of Medicine, University of Freiburg, Freiburg, Germany

**Keywords:** Idiopathic Orbital Inflammation, Optic nerve sheath meningioma, Optic pathway glioma, Orbital Lymphoproliferative Lesions, IgG 4-Related Disease, Orbital Metastases

## Case Report

**Fig. 1:**
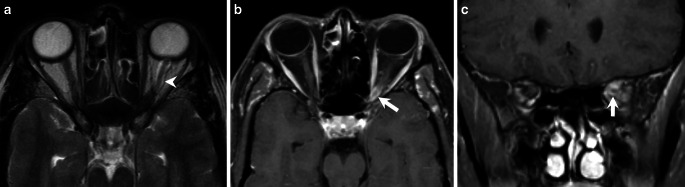
Axial T2-weighted MRI from March 2008 (**a**) revealed a dilatation of the dural space along optic nerve (ON) sheath (arrowhead). Axial (**b**) and coronal (**c**) T1-weighted contrast-enhanced fat-suppressed (T1 C+ FS) images demonstrated a concentric, enhancing lesion encasing the compressed, non-enhancing ON, producing the characteristic “tram-track” and “doughnut” appearances (arrows)

A 60-years-old female patient, first presented in 2008 with a transient visual impairment of the left eye and pathological visually evoked potentials. Magnetic resonance imaging (MRI) revealed a unilateral, concentric, enhancing lesion encasing the non-enhancing left optic nerve sheath (Fig. 1). Cerebrospinal fluid showed a mild pleocytosis with negative oligoclonal bands and no intrathecal immunoglobulin synthesis. In 2011, a second episode of transient visual impairment of the left eye occured, which spontaneously regressed. CSF diagnostics again showed pleocytosis with evidence of oligoclonal bands in the CSF, whereupon immunosuppressive therapy was initiated under the suspected diagnosis of an inflammatory CNS disease. In 2018, the patient presented with an external oculomotor nerve palsy. Imaging revealed a thickened optic nerve sheath and a progressive congestion of the optic nerve sheath over the course of 10 years (not shown). CSF showed persistent pleocytosis. After discussion in the interdisciplinary brain tumour board a local radiotherapy was performed under the suspected diagnosis of an optic nerve sheath meningioma.

During the course of the disease, repeated immunological examinations for vasculitis were carried out, each time without conclusive findings. Serological examinations with regard to an infectious genesis of the optic neuritis remained unremarkable except for a positive serology for bartonella on two occasions (2008 and 2024). The pathogen diagnostics from the cerebrospinal fluid and the surgical specimen also yielded no conclusive findings so far.

**Fig. 2:**
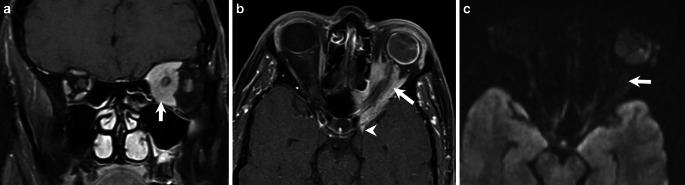
Follow-up MRI of the orbit in November 2024. Coronal T1-weighted contrast-enhanced fat-suppressed (T1 C+ FS) MR image (**a**) demonstrated enhancement of the left orbital mass, which appeared eccentric, suggesting possible growth beyond the confines of the optic nerve sheath. The medial portion encased the non-enhancing optic nerve (arrow). Axial T1 C+ FS image (**b**) showed a contrast-enhancing lesion surrounding the intraorbital and intracanalicular segments of the left optic nerve (arrow), with a dural tail visible along the sphenoid lesser wing (arrowhead). Axial diffusion-weighted images with b 1000 maps revealed no diffusion restriction of the solid tumor mass (arrow)

In the further course the patient suffered complete visual loss of the left eye as well as severe restriction of oculomotor function. Imaging revealed a progressive atrophy of the optic nerve and an increase in size with persistent contrast agent uptake of the optic nerve sheath tumor (Fig. 2) so that the indication for surgical resection was made in 2024.

In light of tumor progression despite prior radiation therapy, and particularly due to imaging findings suggesting early infiltration of the intradural/prechiasmatic ipsilateral optic nerve, an indication for resection was made. Given the complete loss of vision and the failure of all cranial nerve functions in the affected eye, the aim was twofold: to achieve maximum tumor reduction while preventing the disease from spreading to the chiasm, thereby protecting contralateral vision through prechiasmatic transection of the affected optic nerve.

A frontolateral craniotomy was performed, during which the orbital roof was first resected extradurally, and the periorbita was opened along the midline. Intraoperatively, it became evident that although the optic nerve was tumorous and enlarged, it was poorly demarcated from the ocular muscles, with significant adhesion or infiltration of both structures. The optic nerve was transected behind the globe, and the tumor mass originating from the nerve was mobilized within the boundaries that could be established towards the surrounding muscles. The tumor was then excised at the apex of the optic canal.

**Fig. 3:**
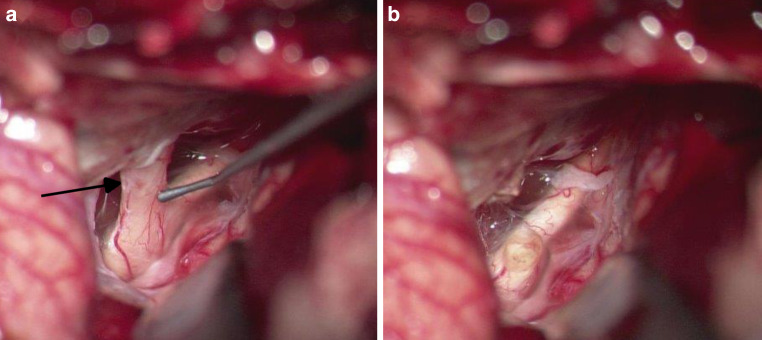
**a** Microscopic view into the intradural space before transection of the left optic nerve showed a questionable infiltration of the pre-canalicular part of the intradural nerve (arrow). **b** The nerve was transected 2 mm distant to the chiasm to preserve potential contralateral fibres looping into the ipsilateral prechiasmatic nerve

Subsequently, the dura was opened, and the optic chiasm was exposed. In the immediate pre-canalicular portion of the ipsilateral optic nerve, microscopic infiltration was suspected (Fig. 3a). Therefore, the optic nerve was transected approximately 2 mm from the chiasm in a microscopically unaffected section (Fig. 3b). Both the periorbita and dura were closed in a watertight fashion.

## Imaging

An MRI obtained upon initial admission in 2008 revealed a unilateral, concentric, enhancing lesion encasing the non-enhancing left optic nerve sheath. The lesion exhibited a clear extension of the dural sheath on T2-weighted images (Fig. 1a, arrow). On axial T1-weighted contrast-enhanced fat-suppressed (T1 C+ FS) MRI, the lesion displayed the characteristic “tram-track” enhancement of the optic nerve sheath, encasing an otherwise normal-appearing optic nerve. Coronal T1 C+ FS images revealed a “doughnut” appearance (Fig. 1b, c, arrows). An axial bone CT showed no calcifications along the intraorbital optic nerve sheaths bilaterally (not shown). At initial diagnosis, there was no evidence of dural thickening, fissural extension, extraocular muscle involvement, or perioptic cysts (typically representing cerebrospinal fluid accumulation proximal to the lesion).

MR imaging in November 2024 demonstrated interval growth of the lesion after a prolonged period of stability. Coronal T1 C+ FS MRI revealed a left orbital mass with marked enhancement, appearing eccentric and suggesting extension beyond the optic nerve sheath (Fig. 2a). The lesion’s medial portion continued to surround the non-enhancing optic nerve. Axial T1 C+ FS MRI showed the lesion encasing both the intraorbital and intracanalicular segments of the optic nerve (arrow), with a dural tail (arrow head)visible along the sphenoid lesser wing (Fig. 2b).

Diffusion-weighted imaging (DWI) revealed no diffusion restriction in the solid, contrast-enhancing parts of the lesion (Fig. 2c, arrow).

## Differential Diagnosis

A wide range of tumors and inflammatory disorders can affect the orbit, with their anatomical location significantly influencing clinical presentation, complications, and treatment strategies. Imaging plays a pivotal role in evaluation, often revealing well- or poorly-marginated, mass-like, or infiltrative lesions with characteristic enhancement and structural changes.

### Idiopathic Orbital Inflammation

Idiopathic Orbital Inflammation (IOI), is a benign, non-infectious inflammatory disorder comprising 8–10% of all orbital masses [1]. IOI can be classified by location into anterior, diffuse, posterior, or apical forms, and further subtypes include myositis, dacryoadenitis, periscleritis, optic perineuritis (OPN), and focal masses [2, 3]. It is typically unilateral, though bilateral involvement occurs in 8–20% of cases [4, 5]. It is most commonly observed in the fifth decade of life and shows no predilection for either sex.

Imaging findings vary depending on the subtype. Lacrimal gland involvement (dacryoadenitis), extraocular muscle enlargement (myositis with tendinous insertion involvement), and retrobulbar extensions are common patterns. CT imaging often reveals irregular, moderately enhancing soft tissue masses, with bone remodeling or erosion in chronic forms. MRI with contrast and fat suppression is the modality of choice, demonstrating hypointensity on T1-weighted images and variable T2-weighted signals, often hypointense in chronic or sclerosing variants due to fibrosis [6, 7].

Optic Perineuritis (OPN) is a rare subtype of IOI, characterized by inflammation of the optic nerve sheath, resulting in significant thickening caused by nonspecific fibrosis [8, 9]. Unlike optic neuritis (ON), which primarily affects the axons of the optic nerve, OPN involves the sheath itself. It is usually idiopathic but may arise secondary to infections or autoimmune conditions such as ANCA-associated vasculitis or sarcoidosis [8]. Imaging findings include poorly marginated, enhancing soft tissue that may appear hypointense on T2-weighted MRI in chronic cases. Diagnostic features include “tram-track” enhancement on axial MRI and “doughnut-shaped” enhancement on coronal MRI views. However, these findings are not exclusive to OPN and may also occur in optic nerve sheath meningioma (ONSM) [2, 8, 10]. Differentiating OPN from ONSM is challenging due to overlapping imaging and clinical features. While ONSM typically presents as a slowly progressive orbitopathy, it can sometimes be distinguished by calcifications visible on CT or optociliary shunt vessels observed during fundoscopic examination. In cases where these distinguishing features are absent, differentiation relies heavily on imaging patterns and clinical context.

In our case, imaging findings were consistent with IOI, possibly of the OPN subtype with a granulomatous histologic variant. However, an initial presumptive diagnosis of meningioma was made based on the clinical presentation and vivid enhancement of the optic sheath. This diagnosis contrasted with the characteristic non-enhancing optic nerve findings, described as the “tram-track sign” on axial images or the “non-enhancing dot sign” on coronal images.

### Optic Nerve Sheath Meningioma

Optic nerve sheath meningioma (ONSM) is a benign, slow-growing tumor originating from the arachnoid cap cells of the optic nerve sheath, accounting for approximately 20% of orbital meningiomas. It typically occurs in the 4th or 5th decades of life, with a female-to-male ratio of 2:1 to 4:1, and juvenile cases are often associated with neurofibromatosis type 2 (NF2) [11, 12]. Clinically, ONSM presents with progressive unilateral vision loss, proptosis, and optic disc pallor or swelling.

Imaging typically shows a circumferential, enhancing mass surrounding the intraorbital optic nerve, with calcifications in 30–50% of cases and characteristic “tram-track” or “doughnut” enhancement patterns. These findings, however, are not pathognomonic [2, 8, 13]. T2-weighted MRI signal varies depending on calcification and histologic subtype. Optic nerve sheath meningioma can be differentiated on the basis of calcifications, which can be seen using CT if the diagnosis is unclear. Perioptic cysts are specific feature and defined as increased cerebrospinal fluid within nerve sheath surrounding distal optic nerve between tumor and globe.

In our case, the presence of an enhancing mass surrounding the intraorbital optic nerve, isointense to grey matter on both T1- and T2-weighted imaging, with vivid enhancement contrasting the non-enhancing optic nerve, supports the diagnosis of ONSM. However, the absence of calcifications on CT makes it challenging to differentiate ONSM from OPN based solely on MRI findings, given the overlapping imaging characteristics.

### Optic Pathway Glioma

Optic pathway glioma (OPG) is a primary neuroglial tumor of the optic pathway, classified into three subtypes: childhood syndromic (associated with neurofibromatosis type 1 [NF1]), childhood sporadic, and adult forms [14–16]. Clinically, OPG may cause decreased vision and proptosis but is often asymptomatic, particularly in childhood. Approximately 30–40% of patients with OPG have NF1, and 11–30% of individuals with NF1 develop OPG [14].

Imaging reveals a fusiform optic nerve mass with variable involvement of the posterior pathway, appearing iso- to mildly hypointense on T1-weighted MRI and variably hyperintense on T2-weighted MRI, with enhancement ranging from minimal to intense. CT is less sensitive than MRI, findings may include enlargement of the optic nerve, with the mass appearing either fusiform or exophytic, as well as enlargement of the optic canal. The optic nerve itself may appear elongated, with features such as kinking or buckling [16].

In our case, the perineural location of the lesion, rather than involvement of the nerve itself, supports a diagnosis of either optic perineuritis (OPN) or optic nerve sheath meningioma (ONSM).

### Orbital Lymphoproliferative Lesions

Orbital lymphoproliferative lesions (OLPL) encompass a spectrum of conditions, ranging from benign lymphoid hyperplasia to malignant lymphoma, most commonly low-grade mucosa-associated lymphoid tissue (MALT) lymphoma. Orbital lymphomas account for only 2% of all lymphomas but constitute 5–15% of all extranodal lymphomas and approximately 50% of all primary orbital malignancies in adults [17].

OLPL most commonly present as an insidious, painless orbital mass, often causing anterior orbital or eyelid swelling. Direct infiltration of the globe or optic nerve is uncommon in lymphoma, and vision is typically preserved. These lesions are most prevalent in older patients (5th–8th decades), with benign forms more common in younger individuals.

These lesions typically present as solid, homogeneously enhancing tumors that mold to and encase orbital structures, often involving the anterior extraconal orbit, particularly the lacrimal gland. They are typically unilateral in primary lymphoma (60–75%) but bilateral in benign OLPL (50–80%). CT shows isodense to slightly hyperdense masses with moderate, homogeneous enhancement, while MRI reveals mild hyperintensity on T1 and T2, low ADC values (< 0.8 × 10⁻^3^ mm^2^/s) in lymphoma, and strong, homogeneous post-contrast enhancement. MRI with contrast is the preferred imaging modality, while CT is commonly the first study performed. Distinguishing features include enhancement patterns, ADC values, and involvement of adjacent structures, with bone destruction indicating aggressive histology [18].

In the present case, despite observing a progressively enlarging mass with homogeneous contrast enhancement encasing the optic nerve and extending in a perineural pattern through the optic canal during follow-up, the slow-onset proptosis and decreased vision, along with the absence of diffusion restriction within the lesion, made a diagnosis of lymphoma less likely.

### IgG 4-Related Disease

IgG 4-Related Disease (IgG 4-RD) is a systemic fibroinflammatory condition affecting various organs, including the orbit, CNS, and head and neck. Orbital involvement commonly presents as painless periorbital swelling, proptosis, and occasionally diplopia [19, 20].

CT and MRI typically show bilateral enlargement of the lacrimal gland and/or extraocular muscles, most often the lateral rectus, with sparing of the anterior tendon. Additional findings may include dura-arachnoid thickening or pituitary and cranial nerve infiltration. MRI reveals isointense lesions on T1-weighted imaging, hypointense on T2, and uniform post-contrast enhancement. IgG 4-RD often overlaps with other infiltrative orbital disorders. For example, thyroid-associated orbitopathy causes tendon-sparing extraocular muscle enlargement but rarely involves the lateral rectus early in the disease or affects the infraorbital nerve. Similarly, idiopathic orbital inflammation is more likely to present with unilateral involvement, anterior tendon involvement, and preferential enlargement of the medial rectus [19, 21].

In our case, the absence of bilateral involvement, preservation of the extraocular muscles and lacrimal gland, lack of involvement of other cranial or extracranial organs, and the absence of supporting clinical and laboratory findings made this diagnosis unlikely.

### Orbital Metastases

In general, orbital metastases are relatively uncommon, accounting for 2–11% of all orbital neoplasms. They primarily involve extraocular structures, distinguishing them from intraocular metastases such as uveal metastases, which are eight times more prevalent [22]. Diagnosis is usually evident in the context of widespread metastatic disease, offering alternative biopsy targets. However, in isolated orbital metastases, a biopsy may be necessary to confirm the diagnosis [22]. Our patient had no known history of malignancy and did not present with a solitary mass, making the longitudinal tram-track enhancement of the optic nerve sheath atypical for metastasis.

## Histology, Immunohistochemistry and Molecular Analyses

**Fig. 4:**
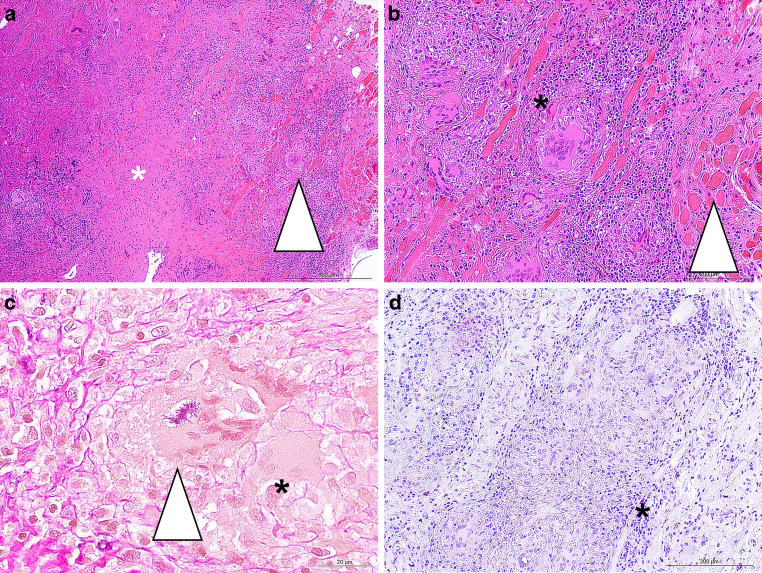
**a** H&E staining showed connective tissue (marked by an asterisk) with perivascular non-ceseating granulomatous inflammation surrounding blood vessels and striated muscle fibers (marked by an arrowhead). The inflammatory infiltrate contained multinucleated giant cells and mixed mononuclear infiltrates. The scale bar represents 500 µm. **b** Higher-magnification image of figure panel a showed multinucleated giant cells (marked by an asterisk) and striated muscle fibers (marked by an arrowhead). The scale bar represents 200 µm. **c** Elastica van Gieson staining showed a giant cell containing an asteroid body (marked by an arrowhead) and one without (marked by an asterisk). The scale bar represents 20 µm. **d** Chloroacetate esterase staining showed sparse granulocyte cells (marked by an asterisk). The scale bar represents 200 µm

Hematoxylin and Eosin (H&E) staining showed mainly connective tissue with non-caseating granuloma (Fig. 4a). Particularly in the perivascular areas, we observed lymphocytic infiltrates (Fig. 4b). Regionally, the enclosed blood vessels appeared obliterated (not shown). Multinucleated giant cells contained so called asteroid bodies (Fig. 4c). These star-shaped structures are not pathognomonic for any disease. Instead, they are commonly observed in granulomatous conditions, including sarcoidosis, foreign body granulomas and tuberculosis [23]. Specialized histological stainings failed to identify Acid-fast bacilli or fungi (not shown). Consistently with an absence of an infection, quite few granulocytes were detected using chloroacetate esterase staining (Fig. 4d).

**Fig. 5:**
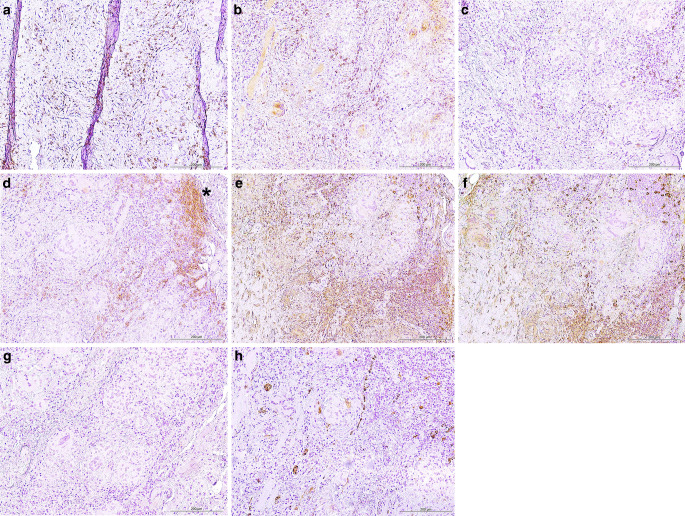
**a** CD3 immunohistochemistry showed numerous T cells within the granulomatous inflammation. The scale bar represents 200 µm. **b** CD8 immunohistochemistry showed that the majority of the T cells within the granulomatous inflammation are cytotoxic T cells. The scale bar represents 200 µm. **c** CD20 immunohistochemistry showed sparse B cells within the granuloma. The scale bar represents 200 µm. **d** CD38 immunohistochemistry showed numerous plasma cells within the granulomatous inflammation. The dense infiltrates are marked by an asterisk. The scale bar represents 200 µm. **e**, **f** Kappa (**e**) and Lambda (**f**) lightchain immunohistochemistry showed that the plasma cells are not lighchain restricted, which would be typical for plasmacytoma. The scale bar represents 200 µm. **g**, **h** Negative CD1a (**g**) and S100 (**h**) immunohistochemistry showed that the lesion does not have markers of histiocytosis. The scale bar represents 200 µm

Next, we utilized immunohistochemistry to further characterize the perivascular infiltrates. We found abundant T cells that mostly consisted of CD8+ cytotoxic cells (Fig. 5a, b). Few, scattered CD20 + B cells were found in lower numbers without emergence of follicular structures (Fig. 5c). Abundant plasma cells were found (Fig. 5d). Absence of light chain restriction made a plasma cell neoplasm unlikely (Fig. 5e, f). Evidence for histiocytosis was not found due to immunohistochemical negativity of CD1a/langerin and S100 as well as lack of histiocytosis-associated histological features (Fig. 5g, h).

In summary, we found a non-caseating granulomatous inflammation without evidence for a neoplastic process.

Potential differential diagnoses included neoplasms, such as meningioma, metastasis, plasmacytoma and histiocytosis, respectively. Infection and foreign body reactions were discussed as exogenous causes of granunulomatosis. Further, systemic disease was discussed as potential underlying cause. Sarcoidosis and granulomatosis with polyangiitis (GPA), formerly Wegener’s granulomatosis, are the main differential diagnoses in that regard. These diagnoses require the identification of systemic disease manifestations. Finally, an exeedingly rare cause of granulomatosis could be idiopathic granulomatous orbital inflammation. This non-specific orbital inflammation represents a diagnosis of exclusion [24]. It would be considered after exclusion of systemic disease and other causes.

Given that we could not find evidence for neoplasia or infectious agents, we chose a descriptive diagnosis.

## Diagnosis

### Connective and Muscle Tissue with Non-caseating Granulomatous Inflammation

The underlying pathology could not be determined from the histological analyses alone. Systemic disease appears as the most likely cause of the lesion. Given the perivascular configuration of the lymphocyte infiltrates with obliterated blood vessels we favour the diagnosis of GPA. The patient is currently undergoing clinical testing for both sarcoidosis and GPA. The tests have not been concluded to date.

Orbital granulomatous lesions are observed in the context of sarcoidosis and GPA [25, 26]. Individual case reports idiopathic granulomatous orbital inflammation have been published [27]. However, this rare diagnosis requires the exclusion of systemic disease.

GPA is an immune-mediated systemic small-vessel vasculitis. The histological presentation includes reactive inflammatory patterns, commonly with necrosis, granulomatous inflammation and vasculitis. Primary affected sites are the respiratory tract and kidneys [27]. The upper respiratory tract with the nasal cavity and paranasal sinuses are common initial disease manifestations. Diagnosis of GPA involves an multidisciplinary approach with clinical assessment, serological testing for anti-neutrophil cytoplasmic antibodies (ANCA) and histological examination.

Sarcoidosis has a wide range of organ manifestations and clinical symptoms [28]. While pulmonary involvement is found in over 90% of cases, virtually any organ can be affected, including ocular and orbital inflammation [29]. Clinical symptoms can be absent or take a dramatic course with organ failure. Diagnosis involves (a) the histological presence of non-caseating granuloma, (b) suggestive clinical presentation and (c) exclusion of other causes of granulomatous lesions [30].

In summary, we present a case of orbital non-caseating granulomatosis. Orbital masses present a common site of manifestation of granulomas. The histological identification requires a multidisciplinary approach to secure the definitive diagnosis of the underlying cause.
